# Leucine-enriched essential amino acid supplementation in mechanically ventilated trauma patients: a feasibility study

**DOI:** 10.1186/s13063-019-3639-2

**Published:** 2019-09-11

**Authors:** L. Wandrag, S. J. Brett, G. S. Frost, M. To, E. Alves Loubo, N. C. Jackson, A. M. Umpleby, V. Bountziouka, M. Hickson

**Affiliations:** 1 Nutrition and Dietetic Research Group, Department of Investigative Medicine, Imperial College London, London, UK; 2 Department of Nutrition & Dietetics, Guy’s & St Thomas’ NHS Foundation Trust, London, UK; 3 Centre for Peri-operative Medicine and Critical Care Research, Imperial College Healthcare NHS Trust, London, UK; 4 Department of Nutritional Science, University of Surrey, Guildford, UK; 5 Statistical Support Service, Population, Policy and Practice Programme, Institute of Child Health, University College London, London, UK; 6 Institute of Health and Community, University of Plymouth, Plymouth, Devon UK

**Keywords:** Muscle wasting, Essential amino acids, Leucine, Muscle ultrasound, Critically ill, Nitrogen balance, Protein turnover, Mechanical ventilation, Trauma

## Abstract

**Background:**

Critically ill patients lose up to 2% of muscle mass per day. We assessed the feasibility of administering a leucine-enriched essential amino acid (L-EAA) supplement to mechanically ventilated trauma patients with the aim of assessing the effect on skeletal muscle mass and function.

**Methods:**

A randomised feasibility study was performed over six months in intensive care (ICU). Patients received 5 g L-EAA five times per day in addition to standard feed (L-EAA group) or standard feed only (control group) for up to 14 days. C-reactive protein, albumin, IL-6, IL-10, urinary 3-MH, nitrogen balance, protein turnover ([1-13C] leucine infusion), muscle depth change (ultrasound), functional change (Katz and Barthel indices) and muscle strength Medical Research Council (MRC) sum score to assess ICU Acquired Weakness were measured sequentially.

**Results:**

Eight patients (9.5% of screened patients) were recruited over six months. L-EAA doses were provided on 91/124 (73%) occasions. Inflammatory and urinary marker data were collected; serial muscle depth measurements were lacking due to short length of stay. Protein turnover studies were performed on five occasions. MRC sum score could not be performed as patients were not able to respond to the screening questions. The Katz and Barthel indices did not change. L-EAA delivery was achievable, but meaningful functional and muscle mass outcome measures require careful consideration in the design of a future randomised controlled trial.

**Conclusion:**

L-EAA was practical to provide, but we found significant barriers to recruitment and measurement of the chosen outcomes which would need to be addressed in the design of a future, large randomised controlled trial.

**Trial registration:**

ISRCTN Registry, ISRCTN79066838. Registered on 25 July 2012.

**Electronic supplementary material:**

The online version of this article (10.1186/s13063-019-3639-2) contains supplementary material, which is available to authorized users.

## Background

Critically ill patients lose up to 2% of muscle volume per day over the first 10 days [1, 2] and physical disability has been identified up to five years after Intensive Care Unit (ICU) admission in severe lung injury patients [3]. Minimising muscle wasting is crucial to optimise recovery. In elderly institutionalised patients, leucine-enriched essential amino acid (L-EAA) supplementation twice daily improved nutritional status, muscle function and physical performance [4]. Leucine supplementation increased muscle protein synthesis in elderly individuals in the short term [5, 6]. This type of supplement has not yet been studied in the critically ill, yet a recent review suggested it warranted further investigation [7].

The aims of this feasibility study were fourfold:

1. To determine the expected rate of recruitment for a future randomised controlled trial (RCT).
2. To establish whether a mechanically ventilated trauma population would represent a feasible patient population to study for this intervention.
3. To determine whether it was possible to routinely deliver a L-EAA supplement in ICU patients.
4. To test the feasibility of measuring muscle mass, protein turnover, strength and physical function outcomes for a future RCT.

## Methods

This prospective randomised feasibility study was conducted at a large London teaching hospital in the adult ICU from May 2012 to December 2012.

Inclusion criteria were: age > 18 years; admission for trauma (head or multiple trauma); expected to be mechanically ventilated for > 48 h; within 72 h of ICU admission. Exclusion criteria were: non-trauma admissions; pregnant or lactating; contraindication to enteral feeding or a pre-existing condition where muscle wasting was exhibited, i.e. patients with congestive cardiac failure, neuromuscular disorders, end-stage renal failure, chronic obstructive pulmonary disease and liver cirrhosis. Trauma patients were selected as these patients are generally younger, fitter and usually present with fewer co-morbidities than other ICU cohorts, thereby representing a more homogenous cohort. The onset of injury can also usually be identified enabling baseline representation of muscle mass and the anticipated length of stay for head or multiple trauma is often significant.

An independent research nurse performed the randomisation of patients via computer-generated schedule to either standard care (control group) or the L-EAA supplement. A branched-chain amino acid ratio for valine : leucine : isoleucine of 1 : 5 : 1 was used based on a previous study where additional leucine was shown to promote muscle protein synthesis [8]. In addition to standard enteral feeding, patients in the treatment group received a 5-g L-EAA powder dissolved in 100 mL of sterile water five times per day via the enteral feeding tube. This was continued for up to 14 days. Control patients received standard enteral feeding via ICU protocol. No placebo was used since at the time of study design the manufacturer could not supply an appropriate product. The study was not blinded as the researcher was also measuring the outcome.

### Study outcome measures

The following measures of feasibility were collected: the number of patients recruited compared with those screened; compliance with administration of the L-EAA dose; and the number and reason for missed outcome measures (inflammatory markers, cytokines, nitrogen balance studies, muscle depth change, protein turnover and functional outcome measures).

Candidate outcome measures to determine rates of muscle wasting, protein turnover, strength and physical function were explored to examine their feasibility in a large RCT. These were muscle depth change on ultrasound, whole-body protein turnover using an intravenous (i.v.) infusion of 1–13C leucine, Katz and Barthel indices to assess functional change and the Medical Research Council (MRC) sum score to assess ICU-acquired weakness (ICU-AW).

C-reactive protein (CRP) (mg/L), albumin (g/L), interleukin (IL)-6 (pg/mL) and IL-10 (pg/mL) levels were measured serially on days 1, 3, 7 and 14 of the study. Cytokines were measured from plasma samples and analysed via enzyme-linked immunosorbent assay (ELISA) using ELISA grade streptavidin HRP and human IL-10 and IL-6 duosets obtained from R&D systems, Oxford, UK.

Muscle depth change (cm) was measured on alternate days throughout the ICU admission. The protocol has been described by Reid et al. [2], but, in brief, includes taking muscle measurements from the bicep, forearm and thigh and adding the measurements together to obtain a total muscle depth (cm). Ultrasound measurements were undertaken using a Sonosite M Turbo™ ultrasound machine with a 5-MHz linear array transducer (Sonosite Ltd., Hitchin, Hertfordshire, UK).

Urinary urea (mmol/24 h) and urinary 3-methylhistidine (μmol/24 h) samples were collected on study days 1, 3, 7 and 14. 3-Methylhistidine (3-MH) i was used as a surrogate marker for skeletal muscle breakdown. An amino acid analyser with cation exchange chromatography (JEOL UK Ltd., Hertfordshire, UK) was used to analyse samples with ninhydrin detection and one inferred standard. Urine urea was measured by a kinetic urease using an Abbott Architect assay with Abbott reagents (Abbott, Maidenhead, UK).

Nitrogen balance (= nitrogen in – nitrogen out) (g/day) was calculated using both the British Dietetic Association’s Parenteral and Enteral Nutrition Group (PENG) recommended equation and the Deacon equation [9] (see Additional file 1).

Katz and Barthel indices were used to assess activities of daily living data. The MRC sum score was used upon awakening and at discharge from ICU as a measure of strength and to determine presence of ICU-AW as recommended by International Guidelines [10].

To measure protein turnover a primed intravenous infusion of 1–13C Leucine 1 mg/kg; 1 mg/kg/h (MassTrace, Somerville, MA, US) was administered for 3 h as previously described [11] on days 1, 3, 7 and 14. Regular blood and breath samples were taken for the measurement of the enrichment of alpha-ketoisocaproic acid (α-KIC), the concentration of leucine and the enrichment of expired CO_2_ at – 10 min, 0 min, 150 min (steady state), 160 min, 170 min and 180 min. Blood samples were taken from indwelling arterial catheters and breath samples were collected from the expiratory port of the ventilator (Servo 300, Siemens, Berlin, Germany or Servo I, Maquet, Sweden) via a Douglas bag with flow meter attached. Enteral feed was stopped 5 h before and during the 3 h of the protein turnover study (see Additional file 1 for protocol and laboratory analysis).

Continuous variables are shown as median (95% confidence interval) due to small sample size; categorical variables are shown as frequencies (%). Descriptive statistics were performed using SPSS V.20 (IBM Corp. 2011).

## Results

**Fig. 1:**
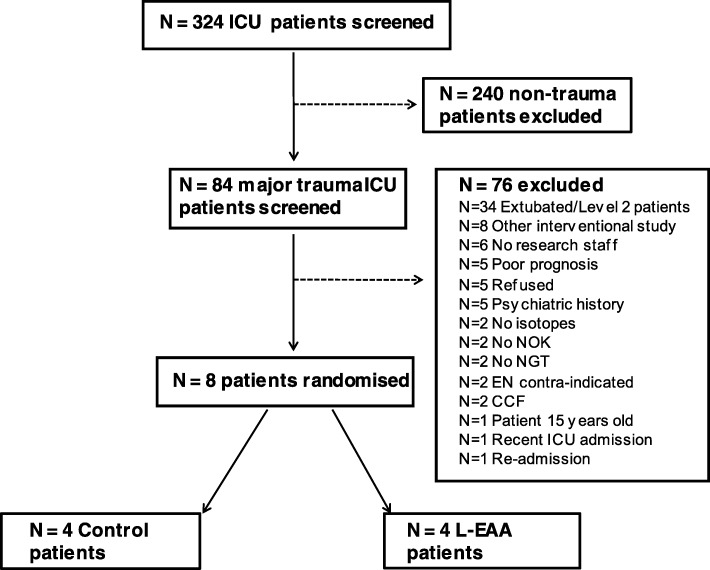
Patient recruitment for the feasibility study. CCF: congestive cardiac failure; EN: enteral nutrition; ICU: intensive Care Unit; L-EAA leucine-enriched essential amino acid, NGT nasogastric tube, NOK next-of-kin. No research staff --> no reserach staff for recruitment and enrolment; recent ICU admission- other hospital; re-admission - same hospital ICU

From 1 May 2012 until 31 December 2012, 324 patients were admitted to intensive care. Eighty-four patients (25.9%) were major trauma admissions and 8 (9.5%) of these patients were recruited to this feasibility study. Patient recruitment is illustrated in Fig. 1.

Demographic data are summarised in Table 1.

**Table 1:** Demographic data of trauma ICU patient cohort

Demographic factors	Control (*n* = 4)	L-EAA (*n* = 4)
Age (years)	60 (28–99)	53 (19–58)
Sex, n (% men)	2 (50)	4 (100)
BMI (kg/m^2^)	23 (20–28)	25 (21–28)
APACHE II (0–71) ^a^	14 (13–20)	15 (11–22)
Injury Severity Score (0–75) ^b^	27 (13–34)	42 (20–50)
Diagnosis, n (%)		
Multiple trauma (no TBI)	1 (12.5)	1 (12.5)
TBI only	1 (12.5)	1 (12.5)
Trauma including TBI	2 (25)	2 (25)
ICU LOS (days)	5 (2–6)	10 (8–14)
Hospital LOS (days)	15 (4–29)	14 (8–30)

*L-EAA* leucine-enriched essential amino acid, *IQR* interquartile range, *BMI* body mass index, *APACHE II* Acute Physiology and Chronic Health Evaluation II, *TBI* traumatic brain injury, *ICU* Intensive Care Unit, *LOS* length of stay

Continuous variables shown as median (95% confidence intervals); categorical variables shown as frequencies (%)

^a^Higher APACHE I scores indicate greater disease severity and correspond with risk of death

^b^Injury Severity Score > 15 is defined as major trauma

The feasibility of data collection is described in Table 2. Muscle ultrasound and protein turnover data collection were much lower than anticipated because patients were transferred back to referring hospitals earlier than anticipated. The Katz and Barthel indices remained at zero for all patients throughout the study and are considered inappropriate for future use in ICU populations. Manual strength testing could not be performed as patients were not able to respond appropriately to the screening questions.

**Table 2:** Feasibility of outcome measures, planned versus achieved measurements

Outcome measure	Target (%)	Achieved (%)	Comment	Control (*n* = 4) Results as range		L-EAA (*n* = 4) Results as range
Patients recruited	> 30	9.5% patients recruited	Lower than anticipated	–		–
L-EAA supplement	> 70	73% doses given	Unable to give due to GI intolerance	–		–
Inflammatory markers and cytokines	> 90	100% samples taken	All required samples were taken	CRP (mg/L)	2–312	42.7–262
				Serum albumin (g/L)	22–30	29–39
				IL-6 (pg/mL)	11.4–141	9–78.5
				IL-10 (pg/mL)	0–137	0–2100
Urine samples (u-urea, 3-MH)	> 80	81% samples taken	CRRT expected in some patients hence > 80% planned No misplaced samples	Urinary urea (mmol/24 h)	55–269	51–382
				3-MH (μmol/24 h)	174–616	144–660
Muscle depth via ultrasound (sum of three sites, planned measurement on alternate days)	> 90	50% measurements completed	Shorter LOS than anticipated Fewer measurements recommended, i.e. days 1, 7, 10	Muscle depth (cm)	5.34–12.36	4.43–11.93
Protein turnover study	> 80	31% studies completed	Complex, time-consuming Required 3 staff members for Douglas bag breath collection method and blood sampling. Simpler technique required, whole body vs muscle protein turnover	Protein turnover (g/24 h)	0	−3.9 to −1.73
Nitrogen balance (PENG, g/day)	> 80	81% calculated	CRRT affecting urine sampling, urinary urea	− 10.6 to 7.4		− 7.95 to 11
Nitrogen balance (Deacon, g/day)	> 80	81% calculated	CRRT affecting urine sampling, urinary urea	− 6.2 to 7.4		− 3.3 to 11.2
Katz and Barthel indices	> 90	100% tested	Poor tests in this setting with substantial floor effect	0		0
MRC sum score	> 90	0% completed	No patient passed initial MRC screening questions (8/10 presented with traumatic brain injury)	–		–

*CRP* C-reactive protein, *IL-6* interleukin 6, *IL-10* interleukin 10, *L-EAA* leucine-enriched essential amino acid, *GI* gastrointestinal, *CRRT* chronic renal replacement therapy, *3-MH* 3-methylhistidine, *LOS* length of stay, *MRC* Medical Research Council, *RFCSA* rectus femoris cross-sectional area

## Discussion

This study was designed to investigate the feasibility of undertaking a RCT providing a L-EAA supplement to critically ill trauma patients to determine the effect on muscle wasting and protein turnover. We found significant barriers to recruitment and to measurement of the chosen outcomes which would need to be addressed in the design of a future, large RCT. Recruitment was dependent on one researcher without 24 h availability. We also found the measurement of some outcomes and frequency thereof to be very intensive for each patient.

Specific recommendations for a future study include the following:

In terms of recruitment, a multicentre design should be considered, potentially as part of a trial group that enables enrolment in multiple sites nationally and/or internationally. The design of a future protocol should allow co-enrolment with other studies, where feasible. We would advise having multiple trained members of staff available to recruit over 24 h, for seven days per week.

In terms of the population studied, the trauma population had some theoretical advantages, fewer co-morbidities, potentially fewer confounders and known onset of injury. However, the dynamic nature of this cohort was underestimated; they had multiple procedures, interventions, admissions to theatre and unexpected repatriation. This made data collection difficult for an intensive physiological study such as this. We would recommend not restricting recruitment to a specific type of ICU patient but including those who are expected to be ventilated for > 48 h and perhaps those patients with high nutritional risk.

Provision of a L-EAA supplement is possible in an ICU setting; however, minimising the number of doses from five times a day to twice daily may aid delivery without reducing any potential beneficial effect. Type and dose of amino acid supplement as well as the best phase of critical illness (acute, chronic, recovery phase) in which to provide this [12] still needs to be explored thoroughly in future studies. Protein supplementation in conjunction with physical activity or exercise may have the best synergistic effect [13]. The current NEXIS (Nutrition and Exercise in Critical Illness) Trial, ClinicalTrials.gov Identifier NCT03021902, in which intravenous amino acids is given alongside in-bed cycle ergometry, will aid to explore this synergistic effect.

In terms of outcome measures, we would suggest that a larger research team is required for such complex outcome measures as stable isotope measurement in ventilated patients. However, the number of staff performing muscle ultrasound measurements should be minimised due to the challenges of achieving good inter-rater reliability. Serial muscle ultrasound measurement over fewer data collection points, i.e. days 1 and 7, may be more sensible. A longer follow-up period would be required in a future amino acid dosing study to assess the post-ICU phase. Patients should be followed up into the ward and after the hospital admission so that longer-term functional outcome measures could be assessed. A mixture of nutritional, physical and functional outcome measures should be included in this type of study. Inflammatory markers and cytokine data were feasible to collect on most occasions. Urinary measurements were only missed due to renal replacement therapy. Serial muscle ultrasound measurement over fewer data collection points, i.e. days 1 and 7, may be more sensible.

The Katz and Barthel indices demonstrated substantial floor effects and were thus poor informers of functional change in critically ill patients. MRC sum score assessment is not advised in a majority head-injured trauma population. Functional assessment tools require piloting in future studies.

To our knowledge, this was the first study to assess the feasibility of using a L-EAA supplement in mechanically ventilated trauma patients. Outcome measures to assess protein turnover, muscle depth change and markers of protein breakdown in conjunction with functional outcome measures are not usually embedded into nutrition trials in the ICU. As such, this study should assist future investigators in considering which ICU patient population and outcome measures to study.

## Conclusion

Amino acid supplementation is feasible in ventilated critically ill patients; however, this study had some major design limitations. Researchers need to consider the study population carefully, perhaps considering individual phenotypes and including patients with high nutritional risk, as well as the detailed clinical pathway. The intervention needs careful consideration for type, dose and timing in terms of phase of illness. Studying interventions both in the ICU and post-ICU phase may be most informative, while coupling amino acids with physical activity. Muscle mass, functional and quality of life outcome measures should be included in future studies.

## Additional file

Additional file 1: Supplementary material [14]. (DOCX 44 kb)

## Data Availability

The dataset used and analysed during the current study is available from the corresponding author on reasonable request.

## Abbreviations

3-MH: 3-Methylhistidine
CRP: C-reactive protein
ELISA: Enzyme-linked immunosorbent assay
ICU: Intensive Care Unit
ICU-AW: Intensive Care Unit-acquired weakness
IL-10: Interleukin 10
IL-6: Interleukin 6
L-EAA: Leucine-enriched essential amino acid
MRC: Medical Research Council
NEXIS trial: Nutrition and Exercise in Critically Illness trial
PENG: Parenteral and Enteral Nutrition Group of the British Dietetic Association
RCT: Randomised controlled trial
α-KIC: Alpha-ketoisocaproic acid
