# Supplementary material for: Leucine-enriched essential amino acid supplementation in mechanically ventilated trauma patients: a feasibility study
Source: Trials. 2019 Sep 11;20:561. doi: 10.1186/s13063-019-3639-2 (PMC6737604; doi:10.1186/s13063-019-3639-2)
Supplement: Supplementary file 1 — Supplementary material [14]. (DOCX 44 kb) [file 13063_2019_3639_MOESM1_ESM.docx]

**Additional file 1**

**Leucine-enriched essential amino acid supplementation in mechanically ventilated trauma patients – a feasibility study**

**Nitrogen balance equations**

British Dietetic Association’s Parenteral & Enteral Nutrition Group equation:

N out: urinary urea (mmol/24h) x 0.033 + obligatory losses. 2-4gN/d (hair, skin, faeces). Add 0.6gN per 1 degree above 37.5.

Deacon equation: Nitrogen excretion (g/24h) = urea excretion (mmol/24h) x 0.028. Add 20% for other urinary losses and a further 2g/day for losses by other routes (9).

Weight (kg), height (m), BMI (kg/m^2^), daily energy (kcal) intake and protein (grams) intake data were recorded. Estimated energy and protein requirements were calculated based on 25-30kcal/kg/day for energy and 1.2g/kg/day for protein (i; ii). Propofol calories were included in calculations. Enteral feed tolerance was monitored by assessing gastric residual volumes (ml) and incidence of vomiting and diarrhoea was also recorded.

ICU demographics were recorded as descriptors of the type and severity of the injury: diagnosis, past medical history, Acute Physiology and Chronic Health Evaluation (APACHE) II scores, Injury Severity Score (ISS), Glasgow Coma Scale, ventilation status, use of renal replacement therapy, ICU length of stay (days), ICU outcome (dead or alive) and hospital length of stay. Steroid use, sedation use, insulin administration (units/24h), fluid balance (ml/24h) and physiotherapy (mobility or rehabilitation therapy) data were collected as factors that may influence muscle integrity.

**IV Leucine stable isotope study**

**Additional file 1 Figure: Stable isotope study protocol**

Alpha-KIC enrichment was measured by gas chromatography and ^13^C enrichment of breath CO_2_ via isotope ratio mass spectrometry as previously described by Whyte *et al* (iii). Measurements of leucine metabolism were calculated using standard isotope dilution equations as described in other studies (11).

References:

(i) Kreymann KG, Berger MM, Deutz NE, Hiesmayr M, Jolliet P, Kazandjiev G, et al. ESPEN Guidelines on Enteral Nutrition: Intensive care. Clin Nutr 2006 Apr;25(2):210-23.

(ii) McClave SA, Martindale RG, Vanek VW, McCarthy M, Roberts P, Taylor B, et al. Guidelines for the Provision and Assessment of Nutrition Support Therapy in the Adult Critically Ill Patient: Society of Critical Care Medicine (SCCM) and American Society for Parenteral and Enteral Nutrition (A.S.P.E.N.). JPEN J Parenter Enteral Nutr 2009 May;33(3):277-316.

(iii) Whyte MB, Jackson NC, Shojaee-Moradie F, Treacher DF, Beale RJ, Jones RH, et al. Metabolic effects of intensive insulin therapy in critically ill patients. Am J Physiol Endocrinol Metab 2010 Mar;298(3):E697-E705.
